# Salivary peptidome profiling analysis for occurrence of new carious lesions in patients with severe early childhood caries

**DOI:** 10.1371/journal.pone.0182712

**Published:** 2017-08-15

**Authors:** Chang Tian, Xiangyu Sun, Xiaochen Liu, Xin Huang, Feng Chen, Shuguo Zheng

**Affiliations:** 1 Department of Preventive Dentistry, Peking University School and Hospital of Stomatology, National Engineering Laboratory for Digital and Material Technology of Stomatology, Beijing Key Laboratory of Digital Stomatology, Beijing, PR China; 2 Central laboratory, Peking University School and Hospital of Stomatology, National Engineering Laboratory for Digital and Material Technology of Stomatology, Beijing Key Laboratory of Digital Stomatology, Beijing, PR China; Virginia Commonwealth University, UNITED STATES

## Abstract

This study aimed to identify differences of peptide profiles in stimulated whole saliva among children with and without occurrence of new carious lesions, and to provide a simple way for early diagnosis and prevention of the relapse of severe early childhood caries (s-ECC). Overall, 26 children aged 3–4 years were selected out from all the children in the kindergarten to be involved in the present study, among them 13 were diagnosed as s-ECC and underwent dental treatment, whilst the other 13 were matched by age and sex as control. Stimulated whole saliva samples were collected before treatment, and at 10 days and 4 months after treatment. During follow-up, 7 of the 13 children with s-ECC showed a relapse, and the new carious lesions were then treated. Salivary peptides were detected using the technique of magnetic beads combined with matrix-assisted laser desorption/ionization time-of-flight mass spectrometry (MALDI-TOF MS). Fifteen peptides showed significant differences in the group without occurrence of new carious lesions (CH group). On comparing the CH group and the other group with occurrence of new carious lesions (CR group), no significant differences were observed before treatment, whereas certain peptides showed significant differences at both 10 days and 4 months after treatment. Two peptides (experimental *m/z* values: 3162.0 Da and 3290.4 Da) exhibited a consistent tendency in cross-sectional and longitudinal comparisons among these groups; these may be associated with recurrence of s-ECC. Based on our findings, it is concluded that different saliva peptide peaks can be detected in s-ECC using MALDI-TOF MS combined with magnetic beads. Moreover, 2 specific peptides with *m/z* values 3162.0 Da and 3290.4 Da could be promising salivary protein biomarkers for diagnosis of recurrence of s-ECC.

## Introduction

Early childhood caries (ECC) is one of the most serious global health concerns among preschool children [1]. Reportedly, according to the 3^rd^ National Oral Health Survey in China, 66% of 5-year-old children had dental caries nationally, and 79.3% of these caries occur in only one-third of all children at this age. After treatment of ECC, recurrence occurs quite early at a high rate, ranging from 22% to 79% [2–4]. Factors that could be associated with continued caries activity in children who had ECC treated include number of extractions (due to fewer numbers of teeth available for relapse), children’s health status, sugar intake frequency, parental compliance with dietary recommendation and oral hygiene guidance, and time of recall appointment [2,5,6]. However, it was found that recurrence had no significant associations with age, gender, race, number of crowns, number of decayed teeth (dt) and salivary *Streptococci Mutans* counts at baseline [2,3]. Considering the high cost and associated long-term damage, it is important to find a way to predict the population that is at high risk of developing ECC.

Whole saliva contains primary saliva, which is secreted in salivary glands, and several of its constituents originate from immune cells, oral microorganisms, mucosal cells, and blood cells [7]. As a result, the multifarious components within saliva can provide clues to oral and systemic diseases [8], including dental caries. Human saliva contains approximately 2,400 proteins, which play important roles in protecting the integrity of dental surfaces [9]. Several of these, including mucins, histatins, albumin, proline-rich peptides, defensins, agglutinins, lactoferrin, and peroxidases, participate in de- and re-mineralization of the enamel, and some of these have been proved to be antimicrobial, which impede the onset of dental caries [10–12].

The success of mass spectrometry (MS) has enabled the discovery and simultaneous analysis of multiple peptide biomarkers, particularly matrix-assisted laser desorption/ionization-time-of-flight mass spectrometry (MALDI-TOF MS) [13]. MS can increase the sensitivity and specificity of the assay and indicate prominent differences between groups [14]. MALDI-TOF MS can detect a large number of proteins and peptides from samples at one time by detecting molecular weight of the analyte MS, without knowing the individual identities of new biomarkers and without the availability of specific monoclonal antibodies. In contrast, the traditionally used enzyme-linked immunosorbent assay (ELISA) uses specific antibodies to indirectly detect biomarkers.

Previous studies have primarily focused on the relationship of salivary peptides and dental caries [15]. However, additional studies are warranted to explore any possible association between salivary peptides and occurrence of new carious lesions in children. The present study aimed to detect the discriminating peptide profiles in saliva of children with and without relapse of severe early childhood caries (s-ECC); this may help us better understand the reason for s-ECC relapse and provide ways for early prevention.

## Materials and methods

### Patient recruitment and sampling

Only children with the following conditions were included: children who were generally healthy, had not received any antibiotics during the past 1 month, were able to spit saliva, agreed with dental treatment and had a complete primary dentition. In total, 26 Chinese children aged 3–4 years (39–49 months) from a kindergarten in the Chaoyang district of Beijing, China, which is the so-called “All-Day Care Kindergarten” in China, were included in this study. All children shared a relatively stable living environment in this kindergarten; they lived in the kindergarten all day long for 5 days a week, consumption planned, trackable, regular menus for their daily meals (3 times a day), and the same way of oral health care such as mouth rinsing after each meal.

Written informed consent was obtained from the parents of all participants included in this study. The study design, protocol, and informed consent details were approved by the Institutional Review Board of Peking University School of Stomatology (No.PKUSSIRB-2013060). All participants were enrolled between December 2014 and November 2015.

From the more than 200 children in the kindergarten, only 13 children could meet all the inclusion criteria shown above and were diagnosed as s-ECC (decayed, missing, and filled tooth surfaces [dmfs] ≥4 for 3-year-olds and dmfs≥5 for 4-year-olds), and they served as the s-ECC group. Other 13 caries-free children were chosen after their age and sex distribution matched with the s-ECC group, hence 26 study participants in total were selected out finally (Table 1). Dental caries was diagnosed according to the World Health Organization caries diagnostic criteria. Caries is recorded as present when a lesion in a pit or fissure, or on a smooth tooth surface, has an unmistakable cavity, undermined enamel, or a detectably softened floor or wall. A tooth with a temporary filling, or one which is sealed but also decayed, should also be diagnosed as caries. Where any doubt exists, caries should not be recorded as present [16]. Only children with no signs of early carious lesions or white spots were designated as caries free. All the participated examiners were experienced clinical dentists, and they had unified training initially in order to make consistent clinical diagnoses. The consistency of the 3 examiners were evaluated prior to clinical examination by examining a small sample (10 children with different caries status) independently for twice, and the overall Kappa value was more than 0.83. Carious lesions were treated using comprehensive restoration and extraction, and children were followed up at around 4 months (closest time to “3 months” follow-up period that the kindergarten and children were available) after treatment. At follow-up, 7 of the 13 children with s-ECC had showed a relapse, and new carious lesions were then treated.

**Table 1:** Demographic and clinical characteristics of participants.

	Without relapse (CH)	With relapse (CR)	Health (H)	p value
	N = 6	N = 7	N = 13	
**Age (months) ± SD**	45.33±3.56	45.57±1.51	45.46±2.82	0.988^a^
**Sex: male**	3 (50%)	3 (42.86%)	9 (69.23%)	1.574^b^
**dmft ± SD**	2.50±0.55	3.14±1.68		0.836^c^
**dmfs ± SD**	6.67±1.21	8.00±4.43		1.000^c^

SD: standard deviation; dmft: decayed, missing, and filled teeth; dmfs: decayed, missing, and filled tooth surfaces

^a^ Analysis of variance (ANOVA)

^b^ Fisher’s exact test

^c^ Mann–Whitney U test

Stimulated whole saliva samples were collected at different time points: before therapy (T1) and at 10 days (T2) and 4 months (T3) after therapy. All children were instructed to avoid taking food or drink for 2 hours before sampling. The samples were collected between 9 a.m. and 11 a.m. Stimulated saliva samples were collected in 5-mL sterile Eppendorf microcentrifuge tubes (Axygen Scientific Inc, USA). These samples were placed on ice immediately and transported to the laboratory within 2 hours. Subsequently, 1 mL of saliva was transferred in a 1.5-mL centrifuge tube and centrifuged at 10,000*×g* for 10 minutes at 4°C; the supernatants (100 μL in a tube) and sediments were collected separately and were stored at −80°C refrigerator until further use.

### WCX fractionation and MALDI-TOF MS

A weak cation exchange magnetic bead (WCX MB) kit was used (Bioyong Tech, Beijing, China). Alpha-cyano-4-hydroxycinnamic acid (CHCA) was dissolved in 100% ethanol (chromatographic grade) and 100% acetone (chromatographic grade) to freshly prepare the sample matrix for MALDI-TOF MS (Bruker Bio-sciences, Bremen, Germany). The suspension in the WCX kit was mixed with samples by shaking. The peptides were separated from the magnetic beads by eluting and beating, and the eluted peptide samples were transferred to a 0.5-mL sample tube (Bioyong Tech). Subsequently, 5 mL of CHCA substrate solution (0.4 g/L, dissolved in acetone and ethanol) and 0.8–1.2 μL of elution peptides were mixed, and 0.8–1.2 μL of this mixture was applied to a metal target plate and dried at room temperature (Bioyong Tech). Finally, the prepared samples were analyzed by MALDI-TOF MS (Bioyong Tech). We used a three-peptide mixture (monoisotopic molecular weights of 1533.8582, 2465.1989, and 5730.6087 Da, Product Numbers P2613, A8346, and I6279, respectively; Sigma-Aldrich, USA) to calibrate the MALDI-TOF MS prior to analyzing our samples. Profile spectra were acquired of 400 shots of laser per sample. A range of 1000–10000 Da peptide molecular weight was detected. Each sample was analyzed thrice, and the mean value of each sample was used for analysis.

### Statistical analysis

The children were divided into 3 groups: without occurrence of new carious lesions 4 months after treatment (CH group, n = 6), with occurrence of new carious lesions 4 months after treatment (CR group, n = 7) and caries free (H group, n = 13). For analysis, p values <0.05 were considered as statistically significant. Mean differences in age among the 3 groups were compared using analysis of variance (ANOVA), and proportional differences in sex were compared using Fisher’s exact test. The nonparametric Mann–Whitney U test was used to compare mean differences among clinical characteristics between the CH and CR groups. Statistical analyses were performed using the SPSS 20.0 software. Based on results of normality tests, ANOVA or Kruskal–Wallis test was used to analyze statistical differences in peptide levels among saliva samples. In addition, data were analyzed using the BioExplorer statistical package (Bioyong Tech).

## Results

No significant differences were observed in the age and sex distribution of children among all groups. Moreover, no significant differences were found in clinical characteristics (i.e., dmft and dmfs) between the CH and CR groups (Table 1).

**Fig 1:**
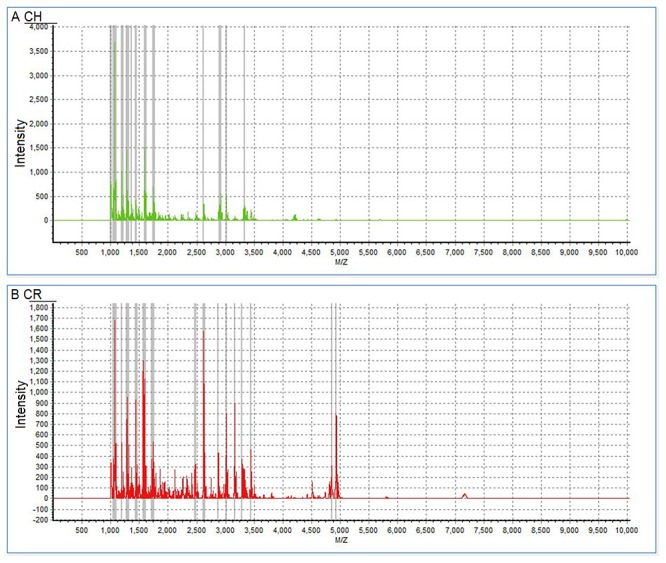
Peptide fingerprints of saliva samples from representative patients. Complete mass spectra of proteins ranging 1,000–7,000 Da show peptide fingerprints of saliva samples from representative patients without occurrence of new carious lesions (A) and with occurrence of new carious lesions (B) at 10 days after treatment.

The entire mass spectra of peptides from the extracted saliva samples from all the 26 participants were analyzed and compared using MALDI-TOF MS. Saliva peptidome fingerprint peaks from each patient were measured based on the maximum intensity within a particular *m/z* range; most peaks were detected in the range of 1,000–7,000 Da (Fig 1). The mass peaks were quantified and compared.

**Fig 2:**
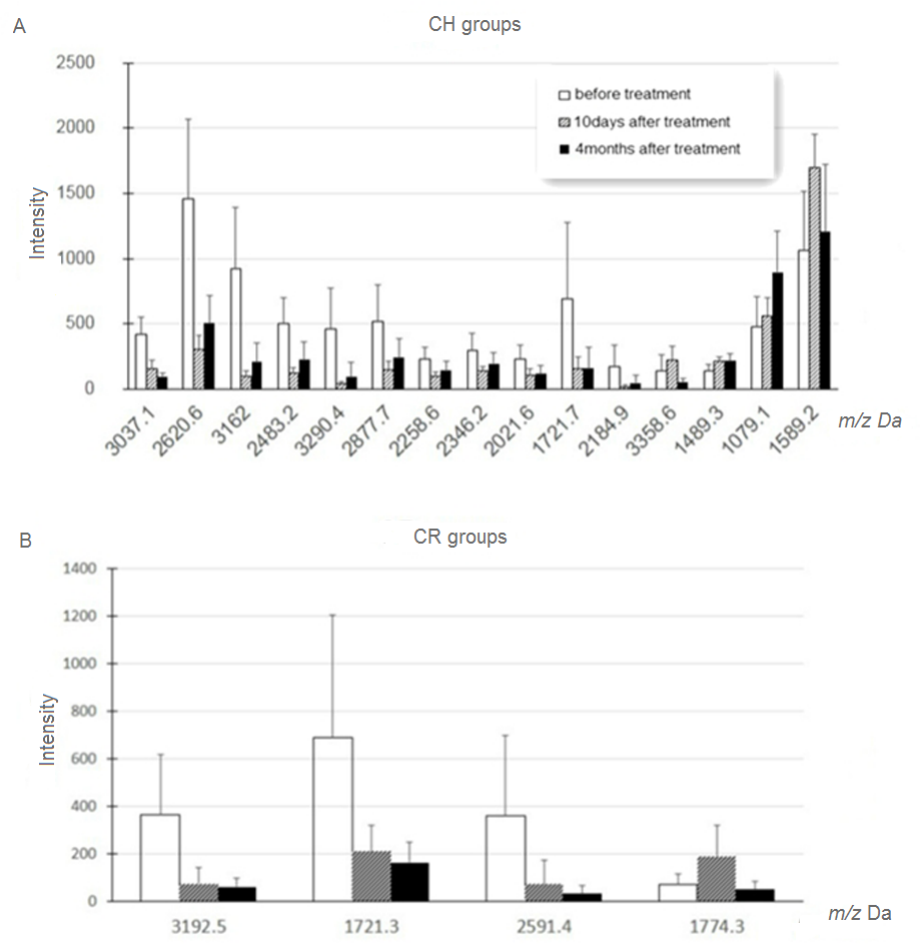
Histogram of mass spectra from the CH and CR groups. Histogram of mass spectra from the CH (A) and CR (B) groups before and after treatment showed increased expression of peptides at 1489.3, 1079.1, 3358.6, and 1589.2 Da and decreased expression of those at 3037.1, 2620.6, 3162.0, 2483.2, 3290.4, 2877.7, 2258.6, 2346.2, 2021.6, 1721.7, and 2184.9 Da in the CH group, and increased expression of peptides at 1774.3 Da and decreased expression of those at 3192.5, 1721.3, and 2591.4 Da in the CR group (p < 0.05).

After removal of peptides that differed significantly among different time points in the control group (caries-free children), a total of 15 peptides in the CH group (3037.1, 2620.6, 3162.0, 2483.2, 3290.4, 2877.7, 1489.3, 2258.6, 1079.1, 2346.2, 2021.6, 1721.7, 3358.6, 1589.2, and 2184.9 Da) and 4 peptides in the CR group (3192.5, 1774.3, 1721.3, and 2591.4 Da) revealed significant differences. In the CH group, 4 peptides (experimental *m/z* values: 1489.3, 1079.1, 3358.6, and 1589.2 Da) were lower before treatment, whereas the other 11 peptides (experimental *m/z* values: 3037.1, 2620.6, 3162.0, 2483.2, 3290.4, 2877.7, 2258.6, 2346.2, 2021.6, 1721.7, and 2184.9 Da) were down-regulated at 10 days and 4 months after treatment (Fig 2A, Table 2). In the CR group, 1 peptide (experimental *m/z* values: 1774.3 Da) was lower before treatment (T1), whereas the other 3 peptides (experimental *m/z* values: 3192.5, 1721.3, and 2591.4Da) were down-regulated at 10 days (T2) and 4 months (T3) after treatment (Fig 2B, Table 3).

**Table 2:** Detected peptides in the CH group at 3 study time points.

*m/z*	p value	T1	T2	T3	Tendency ^a^
**3037.1**	0.00004	418	153.8	94.2	↓
**2620.6**	0.00024	1455	307.8	504.7	↓
**3162.0**	0.00034	919.2	95.7	207.8	↓
**2483.2**	0.001	499.5	120.2	223.3	↓
**3290.4**	0.003	463.5	36	93.3	↓
**2877.7**	0.01	516	145.3	240.8	↓
**1489.3**	0.016	135.2	214.5	214.2	↑
**2258.6**	0.021	226	98.5	141	↓
**1079.1**	0.021	479.8	559.3	893.2	↑
**2346.2**	0.024	298.5	138	194.8	↓
**2021.6**	0.025	230.8	109.2	118.3	↓
**1721.7**	0.017	687.8	159.3	158	↓
**3358.6**	0.03	138	220.3	52.2	↑
**1589.2**	0.047	1061.2	1696	1203	↑
**2184.9**	0.012	170.7	17.8	45.3	↓

^a^ Tendency refers to the trend in the intensity of *m/z* values among the 3 groups

↓ = Higher intensity in the group before treatment (T1) than after treatment (T2 and T3)

↑ = Lower intensity in the group before treatment (T1) than after treatment (T2 and T3)

**Table 3:** Detected peptides in the CR group at the 3 time points.

*m/z*	p value	T1	T2	T3	Tendency ^a^
**3192.5**	0.003	362.9	73	60.4	↓
**1774.3**	0.01	69.4	189.3	49.7	↑
**1721.3**	0.012	689.9	209.2	162.4	↓
**2591.4**	0.026	357.9	70.2	30.9	↓

^a^ Tendency refers to the trend in the intensity of *m/z* values among the 3 groups

↓ = Higher intensity in the group before treatment (T1) than after treatment (T2 and T3)

↑ = Lower intensity in the group before treatment (T1) than after treatment (T2 and T3)

On comparing the CH and CR groups, no significant differences were found before treatment, whereas 8 peptides (2483.2, 1611.6, 3336.5, 3290.4, 3279.6, 3162.0, 3358.6, and 1312.5 Da) revealed significant differences at T2 (Table 4), and 11 peptides (1219.5, 1195.5, 4934.5, 2620.6, 3162.0, 1389.1, 4913.0, 1231.9, 4853.7, 1067.5, and 3290.4 Da) revealed significant differences at T3 (Table 5). On the other hand, compared to CR group, in the CH group, 4 peptides (1611.6, 3336.5, 3358.6, and 1312.5 Da) were higher and 4 (2483.2, 3290.4, 3279.6, and 3162.0 Da) were lower at T2; Moreover, 5 peptides (1219.5, 1195.5, 1389.1, 1231.9, and 1067.5 Da) were higher and 6 (4934.5, 2620.6, 3162.0, 4913.0, 4853.7, and 3290.4 Da) were lower at T3 in the CH group.

**Table 4:** Detected peptides of CH and CR group at T2.

*m/z*	p value	CH	CR	Tendency ^a^
**2483.2**	0.004	120.2	299.7	↑
**1611.6**	0.012	586.7	324.5	↓
**3336.5**	0.014	635.2	233	↓
**3290.4**	0.019	34.3	270.3	↑
**3279.6**	0.024	30.2	259.8	↑
**3162.0**	0.034	95.7	410	↑
**3358.6**	0.036	220.3	103.2	↓
**1312.5**	0.037	609.3	368.5	↓

^a^ Tendency refers to the trend in the intensity of *m/z* values of CH and CR groups

↓ = Higher intensity in CH group

↑ = Lower intensity in CH group

**Table 5:** Detected peptides of CH and CR group at T3.

*m/z*	p value	CH	CR	Tendency ^a^
**1219.5**	0.015	406.8	224	↓
**1195.5**	0.016	1025.3	503.4	↓
**4934.5**	0.025	121.8	816.4	↑
**2620.6**	0.031	504.7	1015	↑
**3162.0**	0.037	206.8	609.3	↑
**1389.1**	0.037	439.2	150.6	↓
**4913.0**	0.039	17.5	186	↑
**1231.9**	0.039	256	115.6	↓
**4853.7**	0.044	20.5	210	↑
**1067.5**	0.044	3659.7	1844.9	↓
**3290.4**	0.044	94.3	302	↑

^a^ Tendency refers to the trend in the intensity of *m/z* values of CH and CR groups

↓ = Higher intensity in CH group

↑ = Lower intensity in CH group

**Fig 3:**
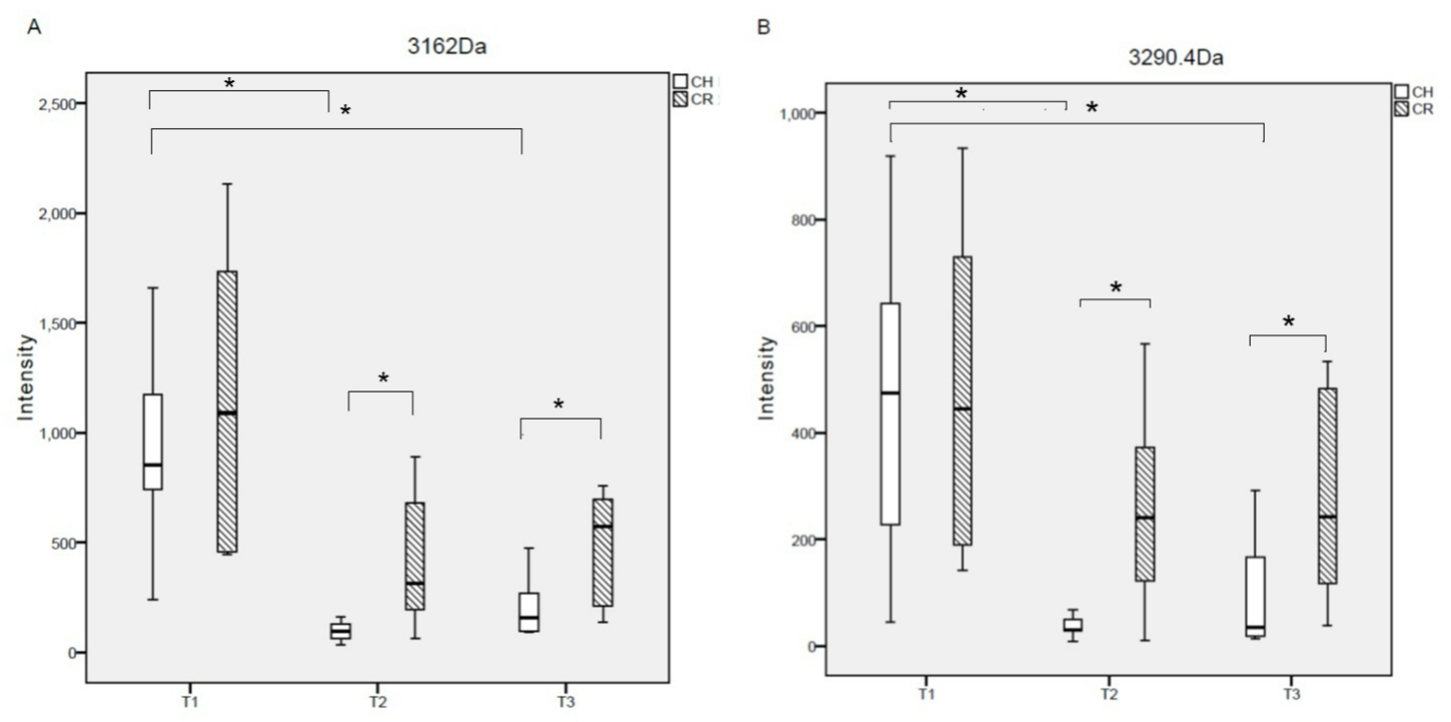
Intensity of *m/z* 3162.0 (A) and 3290.4 (B) for the CH and CR groups. T1 = before treatment, T2 = 10 days after treatment, T3 = 4 months after treatment, *p < 0.05.

**Fig 4:**
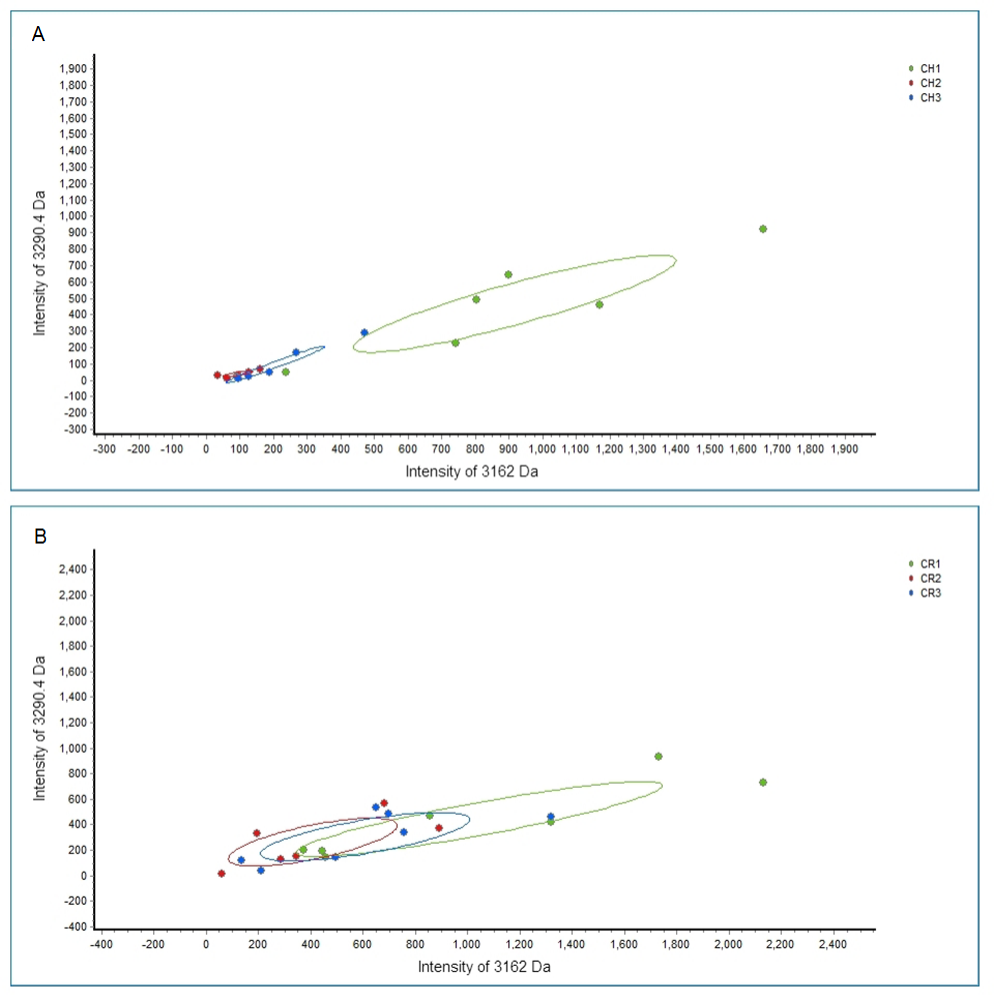
Scatter plots of CH and CR groups. Scatter plots of all 3 groups before and after treatment of children without relapse (A) and those with relapse (B) established by combining the peptides at 3162.0 and 3290.4 Da, showing well-distinguished curve fitting in the CH group.

Two peptides (experimental *m/z* values: 3162.0 and 3290.4 Da) exhibited the same tendency in cross-sectional and longitudinal comparisons across all groups, i.e., these 2 peptides were down-regulated after treatment in the CH group. Similarly, in the CR group, these 2 peptides were down-regulated after treatment, but the difference was not significant (p>0.05). Moreover, at 10 days and 4 months after treatment, these 2 peptides were lower in the CH group than in the CR group (Fig 3). Therefore, we selected these 2 peptides to establish a fitted curve (Fig 4). The shape of the curve showed that samples from the CH group were well separated (Fig 4A), indicating that the fitting results were satisfactory. However, samples from the CR group were not well separated (Fig 4B).

## Discussion

The present study detected and discriminated salivary peptides from children aged 3–4 years with and without recurrence of s-ECC by using the MB-based MALDI-TOF MS technique, aiming to identify a possible association between salivary peptides and the presence/absence of recurrence of s-ECC. Previous studies have associated salivary peptides and human diseases [17–23], and certain studies have demonstrated that salivary peptides may contribute to ECC [24,25]. In this study, on comparing peptides before and after treatment in children from the CH or CR group, we removed the peptides that showed significant differences in the H group, indicating that these different peptides were produced by and depended on treatment instead of age, diet, or other confounding factors. Intensities of the 15 peptide peaks showed significant differences among the pre- and post-treatment CH subgroups (Table 2). Of these, 4 peptides (experimental *m/z* values: 1489.3, 1079.1, 3358.6, and 1589.2 Da) were lower before treatment, whereas remaining 11 peptides (experimental *m/z* values: 3037.1, 2620.6, 3162.0, 2483.2, 3290.4, 2877.7, 2258.6, 2346.2, 2021.6, 1721.7, and 2184.9 Da) were down-regulated 10 days and 4 months after treatment, indicating that peptides can be used to differentiate the status of dental caries.

A previous study reported significant differences in the 17 kDa salivary protein based on varying status of dental caries (active caries and experienced caries) [15]. Moreover, another study reported significant differences in 7 peptide peaks between pre- and post-treatment s-ECC groups [25]. However, in the present study, only 4 peptides differed significantly between the pre- and post-treatment CR subgroups, indicating that salivary peptides of children without a relapse are more sensitive to treatment and that the treatment procedure was more useful in children without a relapse. Furthermore, on comparing the CH and CR groups, no significant differences were found before treatment, whereas the peptides revealed significant differences at both 10 days and 4 months after treatment. At 10 days after treatment, preceding the manifestations of clinical symptoms of the relapse of ECC, we had already observed the differences between children in the CH and CR groups, indicating that peptide changes occurred before the occurrence of new carious lesions and it can be potentially used to predict relapse of dental caries.

It was previously suggested that microbiota development was perturbed by onset of dental caries [26]. We observed 2 peptides (experimental *m/z* values: 3162.0 and 3290.4 Da) that exhibited the same tendency in cross-sectional and longitudinal comparisons among these groups (Fig 3). In both CH and CR groups, these 2 peptides were down-regulated after treatment. However, the differences were not so significant in the pre- and post-treatment CR subgroups. These findings indicate that peptide changes were also disturbed by onset of potential caries. Moreover, these 2 peptides were lower in the CH group than in the CR group at 10 days and 4 months after treatment. Thus, we presumed that these peptides promote the recurrence of ECC and may be associated with the relapse of s-ECC.

Some limitations need to be kept in mind when extrapolating these findings to further application. First, the number of children with s-ECC we analyzed was a bit small, as these children were all that we could found in the kindergarten to be diagnosed as s-ECC and also meet our inclusion criteria. However, as they had a stable living environment all day long in the same kindergarten, we thought that recruiting children from the same kindergarten had advantage of avoiding certain confounding factors. Ideally, further investigation in a larger “All-Day Care Kindergarten” could provide stronger evidence for this issue. Second, the children had only mouth rinsing in the kindergarten without toothbrushing there, leaving their parents to be responsible for toothbrushing as a part of home dental care. Although the children had the same frequency of topical fluoride application by dental professionals in the kindergarten, their fluoride intake at home, e.g. use of fluoride toothpaste, was not clearly known. It would be better if this point could be addressed in our future study. Third, as an “early stage” of dental caries, white spots were receiving more attention by dental professionals to predict potential tooth decay. Though we were using the dental examination methods recommended by WHO [16] and white spots were not recorded in detail in this study, this kind of lesion will surely be taken into account in our future studies if we want to draw a full map of the recurrence of dental caries. Besides, in order to move in further, the in-depth exploration for the source of these peptides could shed some more light on the diagnostic and prediction model of recurrence of s-ECC by detection of saliva, and this would certainly be helpful in improving early diagnosis and surveillance of relapse of dental caries in children.

## Conclusions

The present study demonstrated that salivary peptides were markedly different between children with and without relapse of ECC, preceding the actual manifestation of clinical symptoms. Salivary peptides in children without relapse seemed to be more sensitive to treatment than those in children with relapse. Two peptides (experimental *m/z* values: 3162.0 and 3290.4 Da) here may be diagnostic indicators of recurrence of s-ECC.

## Supporting information

S1 Table Demographic data of all the subjects. (DOCX)

S2 Table Comparison of the 38 peptide peaks detected simultaneously in the H group at the three time points. (DOCX)

S3 Table Comparison of the 34 peptide peaks detected simultaneously in the CH group at the three time points. (DOCX)

S4 Table Comparison of the 11 peptide peaks detected simultaneously in the CR group at the three time points. (DOCX)

S5 Table Comparison of the 8 peptide peaks detected simultaneously in the CH and CR group at T2. (DOCX)

S6 Table Comparison of the 11 peptide peaks detected simultaneously in the CH and CR group at T3. (DOCX)
