# Supplementary material for: Salivary peptidome profiling analysis for occurrence of new carious lesions in patients with severe early childhood caries
Source: PLoS One. 2017 Aug 15;12(8):e0182712. doi: 10.1371/journal.pone.0182712 (PMC5557491; doi:10.1371/journal.pone.0182712)
Supplement: S1 Table — (DOCX) [file pone.0182712.s001.docx]

**Supporting information**

**S1 Table. Demographic data of all the subjects**

| Patient ID | Age (months) | Sex^a^ | dmft | dmfs |
| --- | --- | --- | --- | --- |
| CH1 | 49 | F | 3 | 7 |
| CH2 | 48 | M | 3 | 6 |
| CH3 | 48 | F | 2 | 6 |
| CH4 | 44 | F | 2 | 6 |
| CH5 | 43 | M | 2 | 6 |
| CH6 | 40 | M | 3 | 9 |
| CR1 | 48 | F | 3 | 4 |
| CR2 | 47 | M | 2 | 6 |
| CR3 | 44 | F | 5 | 17 |
| CR4 | 44 | M | 2 | 5 |
| CR5 | 46 | F | 2 | 6 |
| CR6 | 45 | F | 6 | 10 |
| CR7 | 45 | M | 2 | 8 |
| H1 | 47 | F | 0 | 0 |
| H2 | 47 | M | 0 | 0 |
| H3 | 47 | F | 0 | 0 |
| H4 | 47 | M | 0 | 0 |
| H5 | 46 | M | 0 | 0 |
| H6 | 45 | M | 0 | 0 |
| H7 | 44 | M | 0 | 0 |
| H8 | 44 | M | 0 | 0 |
| H9 | 41 | M | 0 | 0 |
| H10 | 39 | F | 0 | 0 |
| H11 | 48 | M | 0 | 0 |
| H12 | 48 | M | 0 | 0 |
| H13 | 48 | F | 0 | 0 |

^a^: F= female; M= male.
