# Supplementary material for: Salivary peptidome profiling analysis for occurrence of new carious lesions in patients with severe early childhood caries
Source: PLoS One. 2017 Aug 15;12(8):e0182712. doi: 10.1371/journal.pone.0182712 (PMC5557491; doi:10.1371/journal.pone.0182712)
Supplement: S2 Table — (DOCX) [file pone.0182712.s002.docx]

**Supporting information**

**S2 Table. Comparison of the 38 peptide peaks detected simultaneously in the H group at the three time points**

| *m/z* | *PTTA(f)* | *P-KWTest* | *PAD_1* | *PAD_2* | *PAD_3* |
| --- | --- | --- | --- | --- | --- |
| 1278.6 | 5.82E-05 | **0.008** | 0.5 | 0.22 | 0.001 |
| 1067.5 | 2.56E-04 | **0.006** | 0.5 | 0.144 | 0.006 |
| 1002.1 | 2.80E-04 | **0.005** | 0.5 | 0.395 | 0.019 |
| 3445.3 | 4.05E-04 | **1.32E-04** | 0.023 | 0.001 | 0.088 |
| 3373.3 | 4.91E-04 | **8.85E-04** | 0.018 | 0.044 | 0.068 |
| 1009.2 | 8.23E-04 | **0.007** | 0.237 | 0.218 | 0.03 |
| 1564.6 | **9.33E-04** | 0.002 | 0.386 | 0.059 | 0.14 |
| 4815.2 | 9.39E-04 | **6.39E-04** | 0.109 | 0.021 | 0.046 |
| 2463.8 | 0.002 | **6.74E-04** | 0.067 | 0.003 | 0.5 |
| 1083 | 0.002 | **0.019** | 0.003 | 0.195 | 0.5 |
| 1612.7 | 0.003 | **0.003** | 0.023 | 0.5 | 0.5 |
| 2642.4 | 0.003 | **0.005** | 0.5 | 0.076 | 0.013 |
| 4553 | 0.003 | **3.93E-04** | 0.031 | 0.006 | 0.02 |
| 1312.5 | 0.004 | **0.002** | 0.5 | 0.493 | 0.001 |
| 3488.2 | **0.004** | 0.042 | 0.432 | 0.122 | 0.101 |
| 4436.7 | 0.005 | **5.08E-06** | 0.001 | 0.159 | 0.024 |
| 1922.4 | 0.006 | **5.38E-04** | 0.088 | 0.004 | 0.007 |
| 3434.2 | 0.006 | **0.004** | 0.066 | 0.001 | 0.021 |
| 3183.7 | 0.006 | **0.004** | 0.214 | 0.03 | 0.001 |
| 4834.9 | 0.006 | **0.009** | 0.216 | 0.031 | 0.147 |
| 1090 | 0.006 | **0.008** | 0.001 | 0.367 | 0.058 |
| 2544.6 | 0.008 | **3.13E-04** | 0.018 | 0.093 | 0.042 |
| 2756.9 | 0.008 | **0.028** | 0.367 | 0.073 | 0.043 |
| 2343.6 | **0.009** | 0.026 | 0.5 | 0.167 | 0.186 |
| 2748.7 | 0.01 | **0.023** | 0.5 | 0.095 | 0.041 |
| 1864.7 | 0.013 | **0.012** | 0.003 | 0.5 | 0.38 |
| 3550.5 | 0.014 | **0.029** | 0.001 | 0.113 | 0.034 |
| 2412.7 | 0.017 | **0.01** | 0.5 | 0.007 | 0.5 |
| 3336.8 | 0.019 | **0.034** | 0.066 | 0.062 | 0.001 |
| 1457.6 | **0.021** | 0.019 | 0.147 | 0.39 | 0.28 |
| 1051.1 | **0.023** | 0.028 | 0.426 | 0.364 | 0.052 |
| 2029.3 | 0.025 | **0.009** | 0.351 | 0.022 | 0.109 |
| 2315.6 | 0.027 | **0.003** | 0.373 | 0.001 | 0.006 |
| 4035.3 | 0.029 | **1.44E-04** | 0.001 | 0.001 | 0.001 |
| 1105.3 | 0.037 | **0.018** | 0.001 | 0.5 | 0.107 |
| 4933.8 | 0.045 | **0.019** | 0.048 | 0.043 | 0.213 |
| 1898.4 | 0.045 | **0.01** | 0.274 | 0.023 | 0.002 |
| 1105.3 | 0.037 | **0.018** | 0.001 | 0.5 | 0.107 |
| 3015.1 | 0.049 | **0.024** | 0.192 | 0.5 | 0.042 |

P<0.05 was considered as threshold of statistical significance.

PTTA(f), P value of ANOVA. P-KWTest, P value of Kruskal-Wallis test.

Which P value was used for the peptide depended on the results of normality tests:

PAD_1, normality test of H group at T1

PAD_2, normality test of H group at T2

PAD_3, normality test of H group at T3
