# Supplementary material for: Salivary peptidome profiling analysis for occurrence of new carious lesions in patients with severe early childhood caries
Source: PLoS One. 2017 Aug 15;12(8):e0182712. doi: 10.1371/journal.pone.0182712 (PMC5557491; doi:10.1371/journal.pone.0182712)
Supplement: S3 Table — (DOCX) [file pone.0182712.s003.docx]

**Supporting information**

**S3 Table. Comparison of the 34 peptide peaks detected simultaneously in the CH group at the three time points**

| *m/z* | *PTTA(f)* | *P-KWTest* | *PAD_1* | *PAD_2* | *PAD_3* |
| --- | --- | --- | --- | --- | --- |
| 3037.1 | **0.00004** | 0.002 | 0.5 | 0.5 | 0.16 |
| 2620.6 | **0.00024** | 0.006 | 0.344 | 0.112 | 0.472 |
| 3162 | **0.00034** | 0.003 | 0.5 | 0.5 | 0.09 |
| 2483.2 | **0.00100** | 0.016 | 0.145 | 0.204 | 0.123 |
| 3290.4 | **0.00300** | 0.018 | 0.5 | 0.5 | 0.098 |
| 2877.7 | **0.01000** | 0.032 | 0.5 | 0.376 | 0.408 |
| 1489.3 | **0.01600** | 0.039 | 0.491 | 0.29 | 0.152 |
| 2258.6 | **0.02100** | 0.042 | 0.5 | 0.05 | 0.5 |
| 1079.1 | **0.02100** | 0.039 | 0.072 | 0.216 | 0.5 |
| 2346.2 | **0.02400** | 0.06 | 0.5 | 0.5 | 0.354 |
| 2021.6 | **0.02500** | 0.078 | 0.171 | 0.437 | 0.5 |
| 1721.7 | 0.03000 | **0.017** | 0.134 | 0.108 | 0.008 |
| 3358.6 | **0.03000** | 0.027 | 0.126 | 0.406 | 0.402 |
| 1589.2 | **0.04700** | 0.103 | 0.085 | 0.5 | 0.297 |
| 2184.9 | 0.04700 | **0.012** | 0.155 | 0.014 | 0.002 |
| *m/z* | *PTTA(f)* | *P-KWTest* | *PAD_1* | *PAD_2* | *PAD_3* |
| 1067.5 | **0.00005** | 7.61E-04 | 0.5 | 0.301 | 0.5 |
| 1002.1 | **0.00036** | 0.004 | 0.383 | 0.274 | 0.5 |
| 2642.4 | **0.00045** | 0.025 | 0.5 | 0.216 | 0.247 |
| 1278.6 | **0.00065** | 0.008 | 0.114 | 0.221 | 0.5 |
| 3183.7 | **0.00069** | 0.023 | 0.5 | 0.5 | 0.309 |
| 4933.8 | **0.00097** | 0.002 | 0.5 | 0.098 | 0.073 |
| 1922.4 | **0.00100** | 0.003 | 0.056 | 0.5 | 0.5 |
| 2748.7 | **0.00100** | 0.003 | 0.5 | 0.226 | 0.059 |
| 1612.7 | **0.00200** | 0.008 | 0.433 | 0.5 | 0.5 |
| 3015.1 | **0.00400** | 0.073 | 0.076 | 0.5 | 0.106 |
| 1564.6 | 0.00700 | **0.011** | 0.171 | 0.253 | 0.021 |
| 1312.5 | **0.00900** | 0.019 | 0.211 | 0.329 | 0.5 |
| 2315.6 | **0.00900** | 0.003 | 0.159 | 0.413 | 0.495 |
| 4815.2 | 0.01000 | **0.003** | 0.5 | 0.014 | 0.111 |
| 1090 | **0.01000** | 0.018 | 0.182 | 0.362 | 0.397 |
| 1457.6 | **0.01300** | 0.027 | 0.5 | 0.5 | 0.5 |
| 3336.8 | **0.02100** | 0.024 | 0.12 | 0.5 | 0.104 |
| 1864.7 | **0.03800** | 0.054 | 0.5 | 0.389 | 0.39 |
| 2463.8 | 0.04600 | **0.031** | 0.001 | 0.122 | 0.073 |

P<0.05 was considered as threshold of statistical significance.

PTTA(f), P value of ANOVA. P-KWTest, P value of Kruskal-Wallis test.

Which P value was used for the peptide depended on the results of normality tests:

PAD_1, normality test of CH group untreated.

PAD_2, normality test of CH group treated for 10 days.

PAD_3, normality test of CH group treated for 4 months.
