# Supplementary material for: Salivary peptidome profiling analysis for occurrence of new carious lesions in patients with severe early childhood caries
Source: PLoS One. 2017 Aug 15;12(8):e0182712. doi: 10.1371/journal.pone.0182712 (PMC5557491; doi:10.1371/journal.pone.0182712)
Supplement: S4 Table — (DOCX) [file pone.0182712.s004.docx]

**Supporting information**

**S4 Table. Comparison of the 11 peptide peaks detected simultaneously in the CR group at the three time points**

| *m/z* | *PTTA(f)* | *P-KWTest* | *PAD_1* | *PAD_2* | *PAD_3* |
| --- | --- | --- | --- | --- | --- |
| 3192.5 | **0.00300** | 0.054 | 0.269 | 0.464 | 0.216 |
| 1774.3 | **0.01000** | 0.045 | 0.372 | 0.253 | 0.311 |
| 1721.3 | **0.01200** | 0.011 | 0.203 | 0.5 | 0.251 |
| 2591.4 | 0.01900 | **0.026** | 0.174 | 0.009 | 0.017 |
| *m/z* | *PTTA(f)* | *P-KWTest* | *PAD_1* | *PAD_2* | *PAD_3* |
| 2315.6 | **0.00200** | 0.009 | 0.455 | 0.5 | 0.5 |
| 3183.7 | **0.00600** | 0.054 | 0.215 | 0.094 | 0.141 |
| 1312.5 | **0.00800** | 0.054 | 0.5 | 0.278 | 0.398 |
| 1922.4 | **0.00900** | 0.015 | 0.5 | 0.5 | 0.5 |
| 1564.6 | 0.01500 | **0.029** | 0.095 | 0.019 | 0.275 |
| 1457.6 | **0.04200** | 0.073 | 0.332 | 0.5 | 0.117 |
| 4815.2 | 0.04300 | **0.044** | 0.16 | 0.011 | 0.5 |

P<0.05 was considered as threshold of statistical significance.

PTTA(f), P value of ANOVA. P-KWTest, P value of Kruskal-Wallis test.

Which P value was used for the peptide depended on the results of normality tests:

PAD_1, normality test of CR group untreated.

PAD_2, normality test of CR group treated for 10 days.

PAD_3, normality test of CR group treated for 4 months.
