# Supplementary material for: Salivary peptidome profiling analysis for occurrence of new carious lesions in patients with severe early childhood caries
Source: PLoS One. 2017 Aug 15;12(8):e0182712. doi: 10.1371/journal.pone.0182712 (PMC5557491; doi:10.1371/journal.pone.0182712)
Supplement: S5 Table — (DOCX) [file pone.0182712.s005.docx]

**Supporting information**

**S5 Table. Comparison of the 8 peptide peaks detected simultaneously in the CH and CR group at T2**

| *m/z* | *PTTA(f)* | *P-KWTest* | *PAD_1* | *PAD_2* |
| --- | --- | --- | --- | --- |
| 2483.2 | **0.004** | 0.002 | 0.204 | 0.054 |
| 1611.6 | **0.012** | 0.041 | 0.5 | 0.5 |
| 3336.5 | **0.014** | 0.041 | 0.5 | 0.349 |
| 3290.4 | **0.019** | 0.041 | 0.5 | 0.5 |
| 3279.6 | **0.024** | 0.041 | 0.5 | 0.5 |
| 3162 | **0.034** | 0.028 | 0.5 | 0.447 |
| 3358.6 | **0.036** | 0.065 | 0.406 | 0.5 |
| 1312.5 | **0.037** | 0.093 | 0.329 | 0.278 |

P<0.05 was considered as threshold of statistical significance.

PTTA(f), P value of ANOVA. P-KWTest, P value of Kruskal-Wallis test.

Which P value was used for the peptide depended on the results of normality tests:

PAD_1, normality test of CH group treated for 10 days (T2).

PAD_2, normality test of CR group treated for 10 days (T2).
