# Supplementary material for: Salivary peptidome profiling analysis for occurrence of new carious lesions in patients with severe early childhood caries
Source: PLoS One. 2017 Aug 15;12(8):e0182712. doi: 10.1371/journal.pone.0182712 (PMC5557491; doi:10.1371/journal.pone.0182712)
Supplement: S6 Table — (DOCX) [file pone.0182712.s006.docx]

**Supporting information**

**S6 Table. Comparison of the 11 peptide peaks detected simultaneously in the CH and CR group at T3**

| *m/z* | *PTTA(f)* | *P-KWTest* | *PAD_1* | *PAD_2* |
| --- | --- | --- | --- | --- |
| 1219.5 | **0.015** | 0.022 | 0.375 | 0.5 |
| 1195.5 | **0.016** | 0.035 | 0.5 | 0.366 |
| 4934.5 | **0.025** | 0.012 | 0.073 | 0.099 |
| 2620.6 | **0.031** | 0.051 | 0.472 | 0.5 |
| 3162 | **0.037** | 0.022 | 0.085 | 0.5 |
| 1389.1 | **0.037** | 0.051 | 0.138 | 0.167 |
| 4913 | **0.039** | 0.014 | 0.138 | 0.055 |
| 1231.9 | **0.039** | 0.066 | 0.491 | 0.222 |
| 4853.7 | **0.044** | 0.014 | 0.092 | 0.079 |
| 1067.5 | **0.044** | 0.073 | 0.5 | 0.167 |
| 3290.4 | **0.044** | 0.051 | 0.1 | 0.264 |

P<0.05 was considered as threshold of statistical significance.

PTTA(f), P value of ANOVA. P-KWTest, P value of Kruskal-Wallis test.

Which P value was used for the peptide depended on the results of normality tests:

PAD_1, normality test of CH group treated for 4 months (T3).

PAD_2, normality test of CR group treated for 4 months (T3).
